# Information capacity and robustness of encoding in the medial prefrontal cortex are modulated by the bioavailability of serotonin and the time elapsed from the cue during a reward-driven task

**DOI:** 10.1038/s41598-021-93313-6

**Published:** 2021-07-06

**Authors:** A. Ezequiel Pereyra, Camilo J. Mininni, B. Silvano Zanutto

**Affiliations:** 1 Present Address: Instituto de Biología y Medicina Experimental (IBYME), CONICET, Buenos Aires, Argentina; 2 Universidad de Buenos Aires, Facultad de Ingeniería, Instituto de Ingeniería Biomédica, Buenos Aires, Argentina

**Keywords:** Computational biology and bioinformatics, Neuroscience

## Abstract

Serotonin (5-HT) is a key neuromodulator of medial prefrontal cortex (mPFC) functions. Pharmacological manipulation of systemic 5-HT bioavailability alters the electrical activity of mPFC neurons. However, 5-HT modulation at the population level is not well characterized. In the present study, we made single neuron extracellular recordings in the mPFC of rats performing an operant conditioning task, and analyzed the effect of systemic administration of fluoxetine (a selective serotonin reuptake inhibitor) on the information encoded in the firing activity of the neural population. Chronic (longer than 15 days), but not acute (less than 15 days), fluoxetine administration reduced the firing rate of mPFC neurons. Moreover, fluoxetine treatment enhanced pairwise entropy but diminished noise correlation and redundancy in the information encoded, thus showing how mPFC differentially encodes information as a function of 5-HT bioavailability. Information about the occurrence of the reward-predictive stimulus was maximized during reward consumption, around 3 to 4 s after the presentation of the cue, and it was higher under chronic fluoxetine treatment. However, the encoded information was less robust to noise corruption when compared to control conditions.

## Introduction

The medial prefrontal cortex (mPFC) is a neocortical area with critical roles in cognition and attention, in top-down processing for guiding behavior and the mapping of sensory stimuli^1^, among other functions^2^. Several studies empathized the role of mPFC in the acquisition of goal-directed learning^3^ and operant response^4^. Electrophysiological recordings showed that populations of neurons of the mPFC respond to reward-related cues by changing their firing rate^5, 6^. The mPFC is a main target of neuromodulator systems^7^, and serotonin (5-HT) is a major modulator of its executive functions^8, 9^.

Serotonergic neurons in the dorsal and medial raphe nucleus (DRN and MRN) are the main source of 5-HT innervation in the mPFC^10, 11^. Serotonergic fibers in mPFC release 5-HT through volume transmission^12^. 5-HT diffuses across mPFC layers and activates several classes of receptors in cell types, such as the inhibitory receptor 5-HT_1A_ and the excitatory receptor 5-HT_2A_, both expressed in pyramidal and GABAergic neurons^13^, and the 5-HT_3_ receptor, mainly found in interneurons^14^. Also, 5-HT receptors are presynaptically expressed, thus modulating the effect of neurotransmitters released in mPFC^15^. Increasing 5-HT availability in mPFC, by local application or electrical stimulation of the DRN, inhibits the spiking of pyramidal cells through 5-HT_1A_ receptors densely located in the axonic cone^16^. Serotonergic input modulates projections from mPFC neurons to neuromodulatory systems and limbic structures through the activation of a variety of receptors^17^, and it also regulates pathways within the PFC, disrupting neural network oscillations^18^. In recent years some evidence emerged demonstrating a role for 5-HT in reward processing^19, 20^. Although mPFC neurons encode reward-related signals and are strongly modulated by 5-HT, there is a lack of studies characterizing its modulation at the circuit level in behaving animals, as well as its involvement in encoding reward-related stimuli^21^.

Here we investigated the neuronal dynamics underpinning the information capacity of the mPFC during reward-associated behaviors and its modulation by the 5-HT bioavailability. We recorded extracellular single-cell electrical activity on the mPFC in vivo in awake rats performing an operant conditioning task in a head-fixed protocol, in order to evaluate the effect of systemic administration of fluoxetine, a selective serotonin reuptake inhibitor (SSRI), on the neuronal population activity. SSRIs are commonly used for the treatment of diseases linked to abnormal serotonergic function, such as anxiety and major depression^22, 23^, which are also related to suboptimal mPFC function^24^. We computed information-theoretical measures, in particular the Shannon entropy and mutual information, together with noise correlation computed across trials for pairs of simultaneously recorded neurons, to determine how mPFC coding of conditioned stimuli is modulated by 5-HT bioavailability. This methodology has the advantage that it makes no assumptions regarding the underlying neural code, and it has been applied to large-scale recordings to understand the nature of the neural population code^25^, the functional interaction between neurons and its role in information processing^26^, and also to study the synergy and redundancy in information representations^27^. Finally, we quantified the robustness of the information conveyed by the population of neurons of the mPFC, and how it is affected under fluoxetine treatment.

## Results

**Figure 1:**
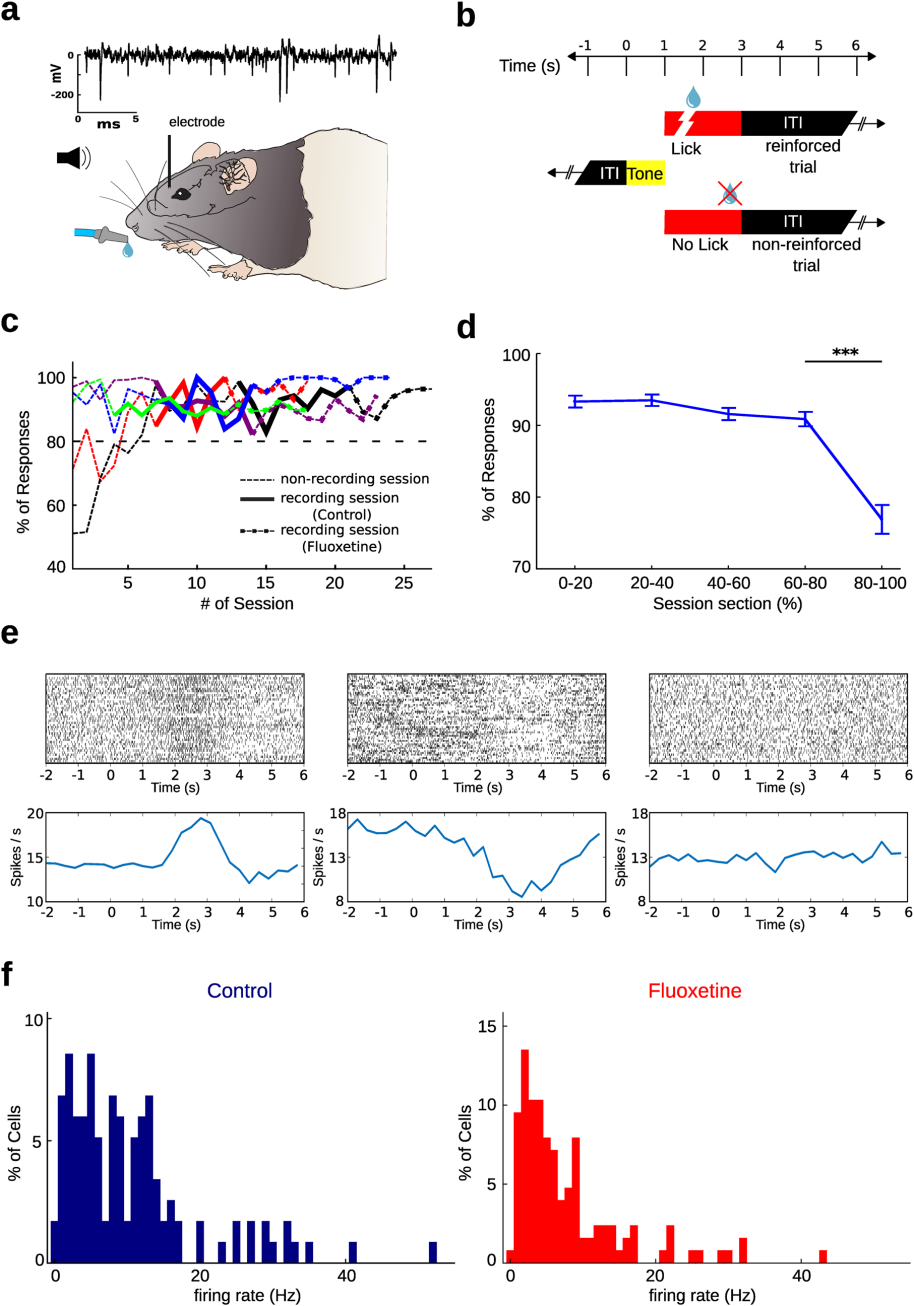
Firing rate profile of neural population recordings in mPFC of behaving rats executing an operant conditioning task. (**a**) Extracellular electrical activity of the neural population was recorded introducing electrodes in the mPFC along the dorso-ventral axis. Rats were implanted with a fixing device in order to maintain the head fixed during the training sessions. An auditory tone, generated by computer, was implemented as a cue. Thirsty animals received a drop of water as reward to a conditioned response. On the top an example of a recording channel is displayed. Electrical signal was high-pass filtered at 300 Hz. (**b**) Training procedure of the operant conditioning task. The auditory cue (tone) was presented for 1 s (yellow bar), followed by an opportunity window of 2 s, in which the rat had to execute the conditioned response (lick) to obtain a drop of water as reward, otherwise the reward was omitted. Both situations were followed by a fixed inter-trial time interval of 10 s. (**c**) Percentage of operant responses per session for each rat (n = 5). Performance of each rat is shown in a different color. Dashed thin lines represent performance in non-recording sessions. Recording sessions of control conditions are shown in thick lines, while recording sessions of fluoxetine condition are shown in dashed thick lines. All rats performed higher than 80% during recording sessions (performance of 80% is marked by a horizontal dotted line). (**d**) Mean percentage of operant responses (± SEM) averaged across all recorded sessions (from all rats) throughout the duration of a session, divided into 5 intervals of equal number of trials. A significant difference was found between the last interval (80–100%) and the previous one (60–80%) (*** p < 0.001, Mann–Whitney U test). (**e**) Top: raster plot illustrating examples of spiking activity of 3 neurons recorded in mPFC during 40 consecutive trials. Bottom: average firing rate (spikes/s) for the same cells. Notice that the first neuron (left) showed an increment in the firing activity with respect to the basal around 3 s after the tone, while the second neuron (middle) showed a decreased firing activity between 3 and 4 s. The third (right) is a neuron with non significant changes in the firing rate. (**f**) Firing rate histograms across the population of recorded neurons under control (left) and fluoxetine (right) treatment. The firing rate of a single neuron was calculated considering only the rewarded trials.

Male *Long Evans* rats were trained to perform an auditory operant conditioning task using a head-fixed paradigm. Single unit population recordings were performed in the medial prefrontal cortex (mPFC) during training (Fig. 1a). Animals had to execute a lick action after the presentation of a tone in order to obtain a drop of water as a reward; otherwise the reward was omitted (Fig. 1b). A total of 68 recording sessions were carried out across five rats that reached the learning criterion: 35 sessions were made under control conditions and 33 sessions under a treatment condition with daily oral administration of fluoxetine (10 mg/kg). All the rats performed, on average, more than 80% of conditioned responses during the recording sessions in both conditions (Fig. 1c). Performance was above 90% during most part of every recording session, and dropped below 80% at the end of the recording sessions (Fig. 1d). Since the percentage of non-rewarded trials was very low (less than 10% of the trials in the first four fifths of the session), we considered only the rewarded trials for further analysis. A total of 117 and 126 neurons, with a signal-to-noise ratio higher than 4, were identified for control and fluoxetine conditions, respectively. Cells recorded from the mPFC exhibited highly variable activity patterns across trials. An inspection of the PSTHs from the recorded neurons showed both increments, decrements or invariant firing rate after the tone compared to their baseline activity (Fig. 1e). This is consistent with previous findings about the effect of reward predicting cues on the activity of mPFC neurons^6^. The distribution of firing rates throughout the population of recorded neurons was similar for both conditions, showing a bias to lower firing rates (Fig. 1f). In control condition the mean firing rate (± SEM) was 10.47 ± 0.86 Hz, while under fluoxetine condition it was 7.56 ± 0.68 Hz, with a significant difference between treatments (p = 0.0042; Mann–Whitney U test). The inhibition of mPFC neuronal activity after administration of fluoxetine is consistent with previous studies carried out on anesthetized rats^32, 33^.

**Figure 2:**
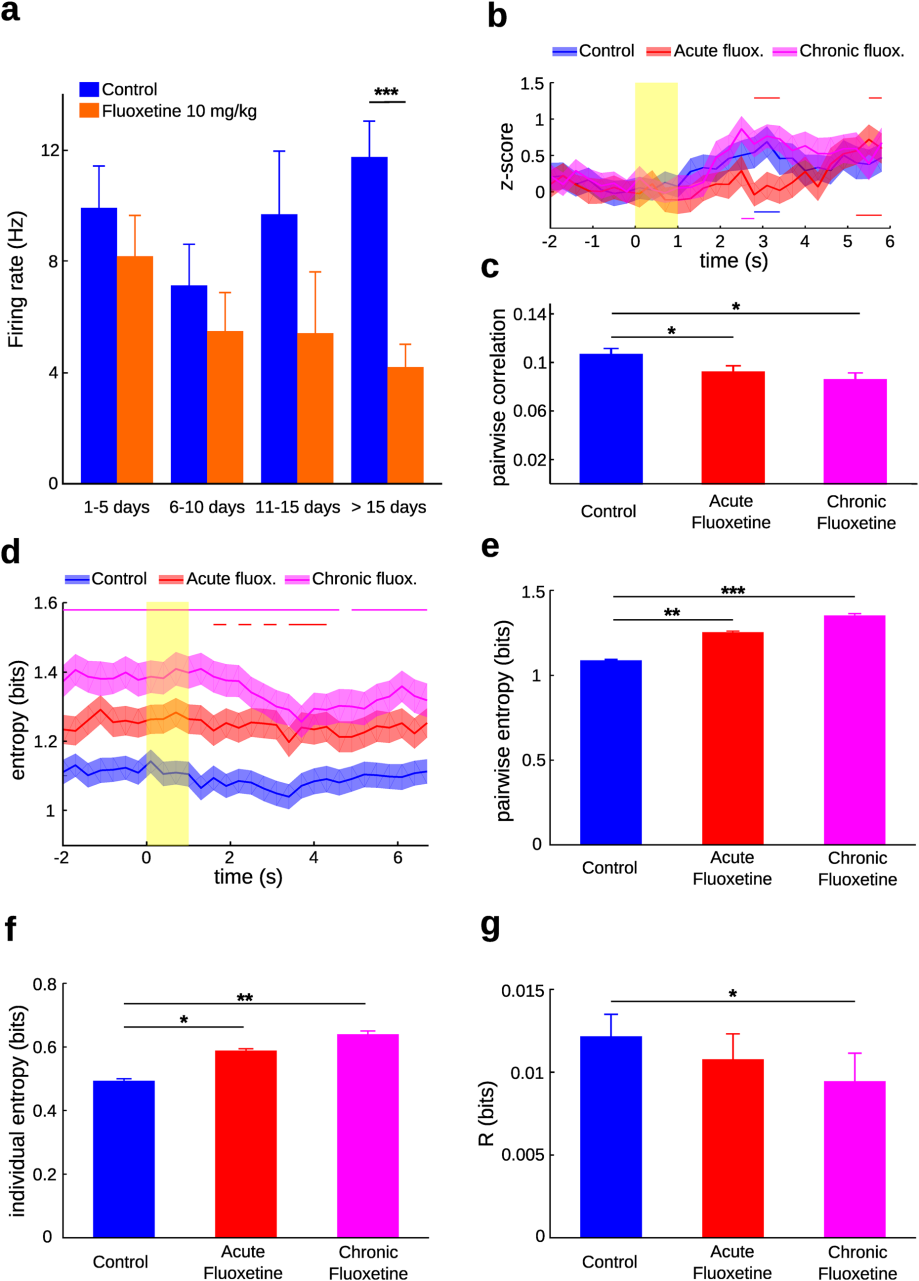
Fluoxetine administration reduced firing rate, decreased noise correlation and increased entropy of mPFC neurons. (**a**) Effect of treatment with fluoxetine on the mean firing activity (± SEM) of mPFC neurons recorded for intervals of 5 days from the first training session in case of control treatment, and from the first daily administration of the drug in case of fluoxetine treatment. Fluoxetine was orally administered at a dose of 10 mg/kg (***p < 0.001, Mann–Whitney U test). (**b**) The z-scores (± SEM) from the averaged firing rate across trials are shown for each treatment using a time window of 300 ms. In order to facilitate visualization, we settled the z-scores to zero at − 0.3 s. Repeated-measures ANOVA showed a significant time by treatment interaction using the Greenhouse–Geisser correction (F (52, 6240) = 1.81, p = 0.03). Multiple comparisons revealed significant differences for each treatment with respect to basal activity (− 1 s ± 0.3 s) for the times indicated with lines on the bottom (p < 0.05, *post-hoc* Tukey's range test comparing time windows with basal values by treatment). Red lines at the top indicate significant differences between control and acute fluoxetine treatment at those times (p < 0.05, *post-hoc* Tukey's range test comparing treatments by time). The time window of the tone is displayed in yellow. (**c**) Mean pairwise correlation (± SEM) of simultaneously recorded neurons using a scale window of 300 ms, for all treatments. The number of paired simultaneous recorded neurons per treatment were: 218 for control, 135 for acute fluoxetine and 91 for chronic fluoxetine. Acute fluoxetine treatment (0.092 ± 0.006) significantly reduced noise correlation compared with control (0.1065 ± 0.005; * p = 0.05, Mann–Whitney U test). Also, chronic fluoxetine treatment (0.0858 ± 0.006) significantly reduced noise correlation compared with control (*p = 0.017, Mann–Whitney U test). We did not find a significant difference between acute and chronic fluoxetine treatments (p > 0.05). (**d**) Pairwise entropy (± SEM) for rewarded trials on each treatment. A time window of 300 ms was employed. The duration of the tone is displayed in yellow. Repeated-measures ANOVA showed a significant time by treatment interaction (F (58, 12789) = 6.51, p < 0.001, using the Greenhouse–Geisser correction). Lines on the top show significant differences between treatments on those times (magenta = chronic fluoxetine compared with control; red = acute fluoxetine compared with control; p < 0.05, *post-hoc* Tukey’s range test comparing treatments by time). Non-significant differences were found on each treatment at times after the tone compared to basal activity (− 1 s ± 0.3 s) (p > 0.05, *post-hoc* Tukey's range test comparing time windows with basal values by treatment). (**e**) Mean pairwise entropy (± SEM) for simultaneously recorded neurons using a timescale of 300 ms are shown for all treatments. Acute fluoxetine treatment (1.252 ± 0.007 *bits*) significantly increased entropy compared with control (1.0863 ± 0.007 *bits*; ** p = 0.003, Mann–Whitney U test). Likewise, chronic fluoxetine treatment (1.350 ± 0.011 *bits*) significantly increased entropy compared with control (*** p < 0.001, Mann–Whitney U test). We did not find a significant difference between acute and chronic fluoxetine treatments (p > 0.05). (**f**) Mean individual entropy (± SEM) for recorded neurons using a timescale of 300 ms are shown for all treatments. Acute fluoxetine treatment (0.588 ± 0.006 *bits*) significantly increased entropy compared with control (0.493 ± 0.006 *bits*; *p = 0.0336, U Mann–Whitney test). Likewise, chronic fluoxetine treatment (0.639 ± 0.010 *bits*) significantly increased entropy compared with control (**p = 0.045, U Mann–Whitney test). We did not find a significant difference between acute and chronic fluoxetine treatments (p > 0.05). (**g**) Mean redundancy (± SEM) for recorded neurons using a timescale of 300 ms are shown for all treatments. Chronic fluoxetine treatment (0.0094 ± 0.0017 *bits*) significantly augmented the measure of redundancy (*R*) compared with control (0.0122 ± 0.0013 *bits*; *p = 0.012, U Mann–Whitney test). We did not find significant differences for the acute fluoxetine treatment (0.0108 ± 0.0016 *bits*) compared with control and the chronic treatment (p > 0.05).

In order to evaluate whether the inhibitory effect of the drug on firing activity was enhanced throughout the days of treatment, neurons were sorted in intervals of 5 consecutive days. The first day of administration of fluoxetine was considered the “day 1” of this treatment. For the control condition the “day 1” was the day of the first recording session. We did not find significant differences in the mean firing rate between successive intervals within each treatment. However, there was a significant difference between treatments for the recordings made after the day 15 (p = 0.00091; Mann–Whitney U test), but not for previous days (Fig. 2a). This result demonstrates that systemic fluoxetine induces an inhibitory effect on the firing rate of mPFC neurons of behaving rats during an operant conditioning task. Moreover, this inhibition is significantly stronger for the chronic treatment when compared with the acute treatment. Based on this result, neurons recorded during fluoxetine treatment were divided in two groups: the acute fluoxetine treatment (neurons recorded up to the fifteenth day of treatment with the drug) and the chronic fluoxetine group (neurons recorded after the fifteenth day of treatment with the drug). The subsequent analyses were computed on neurons from the control (n = 117), acute fluoxetine (n = 76), and chronic fluoxetine (n = 50) treatments.

We observed significant variability in the firing of individual neurons after the tone (see Supplementary Table S1). To tackle this variability we normalized the firing rate by computing their z-scores from − 2 to + 6 s, taking zero as the time of tone onset. Repeated-measures ANOVA revealed a significant treatment by time window interaction (F (52, 6240) = 1.81, p = 0.03, using the Greenhouse–Geisser correction). Both control and chronic fluoxetine neurons increased their z-score around 3 s after the conditioned stimuli compared to basal activity, whereas acute fluoxetine neurons increased their z-score between + 5 and + 6 s (p < 0.05, *post-hoc* Tukey's range tests comparing time windows with basal values by condition). Also, neurons in the control and chronic fluoxetine conditions fired more than the acute fluoxetine group during their peak (p < 0.05, *post-hoc* Tukey's range tests comparing conditions by time).

Subsequently, we applied information theoretic measures in order to determine how much information neurons carried in their firing pattern under each treatment. We measured pairwise entropy and noise correlation of simultaneously recorded neurons of mPFC, computed on fluctuations of the firing rates around the average across trials. These computations were implemented in the transformed binary data using a fixed time window (see “Materials and methods”). On average, fluoxetine treatment significantly decreased pairwise noise correlation. Differences in the mean noise correlation (± SEM) of the acute (0.092 ± 0.006) and chronic (0.0858 ± 0.006) fluoxetine treatment were statistically different respect to control (0.1065 ± 0.005; p = 0.05 and p = 0.017 respectively (Mann–Whitney U test, Fig. 2c). The detrimental effect of the drug on pairwise correlation was progressive, although we did not find a significant difference between the acute and chronic treatment (p > 0.05; Mann–Whitney U test). Figure 2d displays the pairwise entropy from − 2 to 7 s in windows of 300 ms. Averaged pairwise entropy across trials reached a minimum before the 4 s from tone onset in all treatment. Repeated-measures ANOVA revealed a significant treatment by time window interaction (F (58, 12,789) = 6.51, p < 0.001, using the Greenhouse–Geisser correction) for pairwise entropy. However, changes in entropy with respect to the basal values (around 1 s before the tone) were only significant for the chronic treatment with the drug, between + 3.7 and + 4 s. Neurons recorded under fluoxetine treatment showed significantly higher entropy values compared to control for almost all times around stimuli presentation (p < 0.05, *post-hoc* Tukey's range tests comparing conditions by time, Fig. 2d). This result is shown more clearly in Fig. 2e, which displays the averaged entropy for all pairs of simultaneously recorded units. Differences in the mean pairwise entropy (± SEM) of the acute (1.252 ± 0.007 bits) and chronic (1.350 ± 0.011 *bits*) fluoxetine treatment were significant respect to control (1.0863 ± 0.007 bits; p = 0.003 and p = 2.93 × 10^–5^ respectively; Mann–Whitney U test; Fig. 2e). Individually, mPFC neurons also showed an increase in entropy with fluoxetine. The mean individual entropy (± SEM) for acute (0.588 ± 0.006 bits) and chronic (0.639 ± 0.010 bits) fluoxetine treatment showed significant differences respect to control (0.493 ± 0.006 bits; p = 0.034 and p = 0,045 respectively; Mann–Whitney U test; Fig. 2f).

The entropy of a pair of neurons is upper bounded by the sum of the entropy of each neuron, and is only equal to the sum when neurons are statistically independent. Any departure from independence results in a reduction in the total amount of information that a pair of neurons is able to codify. Therefore, we measured the redundancy (*R*) in encoding capacity as the mutual information computed over pairs of neurons, as shown in Eq. (2) of the “Materials and methods” section. On average, *R* (± SEM) was significantly reduced for the chronic fluoxetine treatment (0.0094 ± 0.0017 *bits*) compared to control conditions (0.0122 ± 0.0013 *bits*; p = 0.012, Mann–Whitney U test) (Fig. 2g). A reduced *R* suggests that fluoxetine treatment is decreasing redundancy in the neural population code, which is consistent with a decreased pairwise correlation, since noise correlation can be regarded as a measure of redundancy in a neural code^34^.

**Figure 3:**
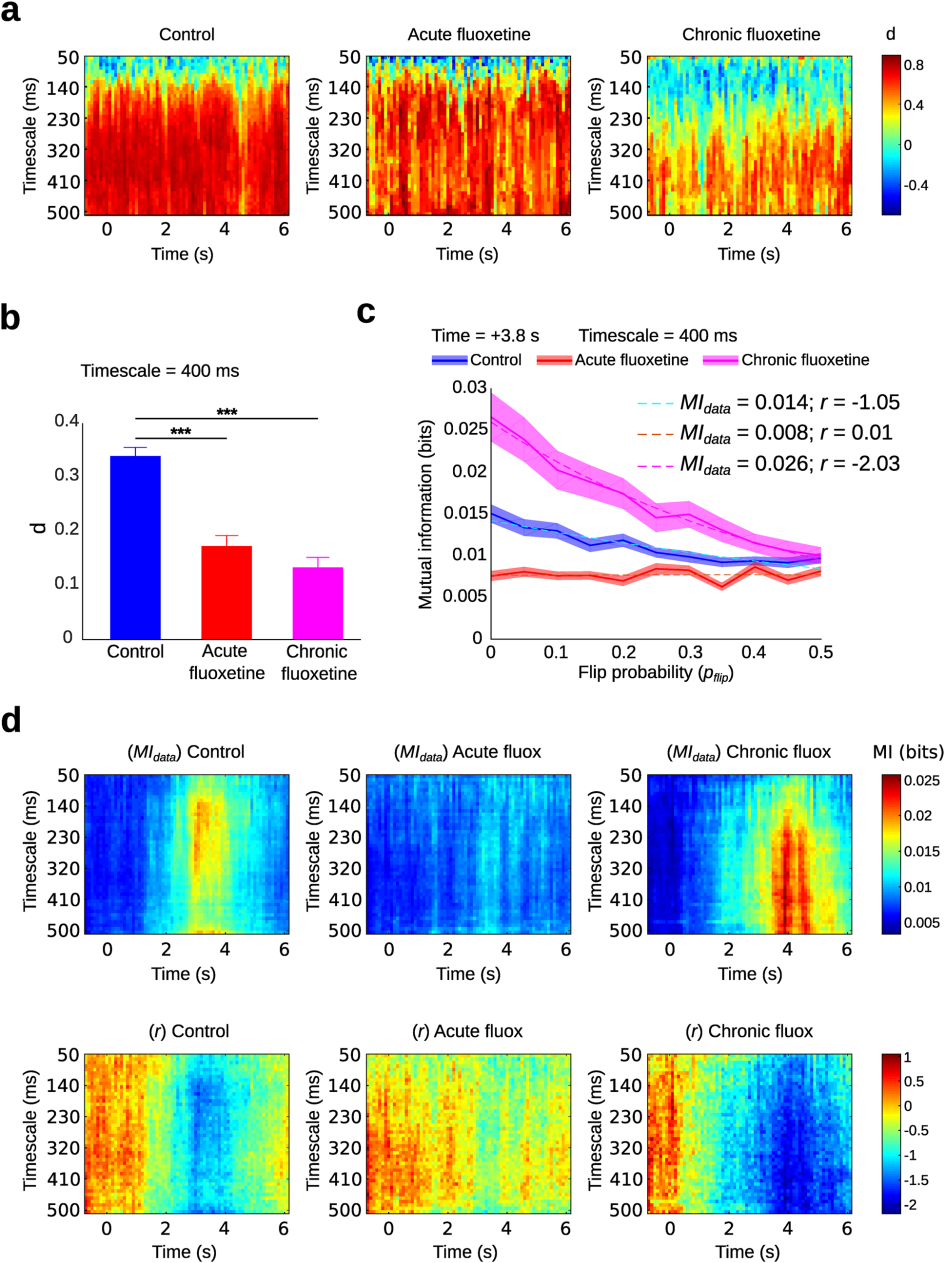
Fluoxetine affected information dependence with noise correlation and impaired robust encoding of the stimulus. (**a**) Dependence (*d*) of pairwise entropy on noise correlation computed as the Spearman’s correlation between measures for several timescales (from 50 to 500 ms) centered at successive times after cue onset (− 1 to + 6 s) for the three treatments. (**b**) Using a timescale of 400 ms the mean Spearman’s correlation between the measures (± SEM), from − 1 to + 6 s, is shown. The mean correlation was reduced for the acute and chronic fluoxetine treatments respect to control group (***p < 0.001, U Mann–Whitney test). We did not find a significant difference between acute and chronic fluoxetine treatments (p > 0.05). (**c**) Example of the exponential fitting curve computed for the pairwise mutual information (MI) as a function of the flip probability (*p*_*flip*_) of the binary state of one neuron of the pair across trials (i.e., state 0 flips to 1, and state 1 flips to 0) using a timescale of 400 ms at 3.8 s after the tone. The intersection point of the estimated exponential function with the y axis (at *p*_*flip*_ = 0) is the uncorrupted MI (*MI*_*data*_). The chronic fluoxetine group of neurons conveyed more information about the occurrence of the stimulus, but it decayed faster with *p*_*flip*_ when compared with the other treatments. (**d**) Parameters *MI*_*data*_ and the robustness factor (*r*) of the exponential fitting curves are shown for several timescales (from 50 to 500 ms) centered at successive times after the tone (− 1 to + 6 s) for the three treatments.

So far, we have found an inverse relationship between average entropy and average correlation of the neural population. Although an increment in entropy is intuitively associated with a decrement in correlation, as shown in Fig. 2, other behaviours are possible, such as an increment or decrement in *both* measures. To gain insight about this issue, we computed the measure *d*, the dependence between entropy and noise correlation, as the Spearman’s correlation coefficient between these two measures, for pairs of simultaneously recorded neurons. As the results depend on the time scale used, we computed *d* for different timescales ranging from 50 to 500 ms. Figure 3a displays the obtained *d* values from − 1 to + 6 s after the tone for several timescales. In general, the dependence was positive, meaning that, in pairs of neurons, entropy and correlation increased or decreased together (Fig. 3a). Treatment with fluoxetine reduced the dependence between entropy and correlation, and for the small timescales, chronic fluoxetine turned this dependence negative. On average, the entropy-correlation dependence, for a window of 400 ms, decreased significantly for acute and chronic fluoxetine treatment compared to control (p < 0.001; Mann–Whitney U test; Fig. 3b). Correlation between neurons can be considered the signature of robust encoding. Thus, the positive dependence between entropy and correlation observed in the control condition suggests that neurons which codify more information do so in a robust manner. This property would be disrupted by the chronic fluoxetine treatment. To further explore this point, we measured how much information neuron activity states conveyed about the occurrence of the stimulus, and compared this information with surrogate activity states that were corrupted by adding noise. Specifically, we assessed the robustness of the encoded information by adding noise to the binary state adopted by one member of the pair across trials (see “Materials and methods”). We computed the mutual information (MI) between basal activity (− 1 s ± timescale) and the activity at a time after stimulus onset, as shown in Eq. (3) of the “Materials and methods” section. Then, the robustness was assessed by computing MI from corrupted neural activity in which neuron states were picked at random and flipped with increasing probability *p*_*flip*_ from 0 to 0.5. Information decayed exponentially fast with flip probability (Fig. 3c), and was well fitted by an exponential curve of the kind: $MI\left({p}_{flip}\right)={MI}_{data}.{e}^{\left(r.{p}_{flip}\right)}$. Parameter *MI*_*data*_ is an estimation of information for uncorrupted activity, while parameter *r* measures the impact of flipping on information, such that higher *r* values reflect higher robustness to noise. Figure 3d shows the values of *MI*_*data*_ (on top panels) and *r* (on bottom panels) of the fitted exponential curves for each treatment, from + 1 s to + 4.5 s after tone onset, and for timescales ranging from 50 to 500 ms. Mutual information was maximized during the opportunity window and afterwards. Interestingly, chronic fluoxetine shifted the maximal MI observed towards longer time windows, and later times from tone onset. Conversely, acute fluoxetine showed the smallest MI values (Fig. 3d, top). Parameter *r* was negatively correlated with *MI*_*data*_, which implies that the higher the information conveyed by the population, the less robust this information was when noise is added. In particular, under chronic treatment with fluoxetine, mPFC neurons conveyed more (but less robust) information about stimuli presentation, while the control condition showed more intermediate values for MI and robustness, consistent with a role of correlation in robust encoding.

In summary, fluoxetine administration, which enhances the systemic bioavailability of 5-HT and alters the normal functioning of the serotonergic system, induced several effects on the firing activity of neurons in the mPFC. In the first place, we observed a reduction in the firing rate of individual neurons. On average, individual and pairwise entropy were enhanced, while noise correlation across trials was diminished after fluoxetine treatment. Furthermore, mPFC encoded information in a less redundant manner in a chronic fluoxetine treatment, suggesting an increased independence between pairs of neurons under this condition. Finally, although chronic drug treatments (longer than fifteen days) allowed mPFC neurons to convey more information about the occurrence of stimuli, it was less robust to noise corruption.

## Discussion

The mPFC has a critical role in the execution of goal-directed behaviours. However, the contribution of serotonergic modulation in mPFC function remains unclear. We found that chronic (more than fifteen days), but not acute or short-term treatment with fluoxetine, a selective serotonin reuptake inhibitor (SSRI), reduces the mean firing rate recorded from mPFC neurons of rats executing an operant conditioning task in a head-fixed protocol. Administration of this drug progressively led to an increment in the mean entropy and a decrement in the mean noise correlation along fluoxetine treatment. In order to interpret our results, we have to make a brief description of fluoxetine effects in the brain. Fluoxetine administration alters the normal functioning of several brain structures. First, the effects of the drug depend on the specialized localization of 5-HT receptors in pyramidal and parvalbumin neurons. As an SSRI, fluoxetine inhibits the 5-HT transporter (5-HTT), increasing the availability of 5-HT in the synaptic and extrasynaptic space in serotonergic targets. 5-HT mainly inhibits pyramidal neurons through activation of the inhibitory receptor type 5-HT_1A_^16^. In the same line, it has been shown that a chronic treatment with fluoxetine enhances the effect of 5-HT on fast-spiking (FS) interneurons in the PFC^35^, which acts via stimulation of the excitatory 5-HT_2A_ receptor. Therefore, a possible neural mechanism that explains the reduced firing rate obtained from recorded mPFC neurons after chronic fluoxetine treatment is an inhibition of pyramidal neurons through the activation of 5-HT_1A_ receptors, or an enhancement of FS interneurons excitability, due to a rise in serotonergic cortical neurotransmission. However, given the systemic nature of our manipulation, the effect of fluoxetine in other brain regions besides the PFC cannot be discarded.

Recorded neurons exhibited heterogeneous temporal spike patterns. Both activations, inhibitions and non significant changes were observed after stimulus presentation (see Supplementary Table S1). To tackle such variability, we take advantage of the Information theory toolset. Specifically, we computed the Shannon entropy, which is a measure that captures the information conveyed in the trial-by-trial fluctuations of the firing rate, making no assumptions regarding the underlying neural code. We observed that, on average, the population of neurons responded within 3 to 4 s after the end of the tone, when we observed that pairwise entropy decreased comitantly with a general increment in the firing rate for all treatments (Fig. 2b,d). A drop in entropy implies a gain in predictability and an attenuation of spiking variability, and is a previously reported phenomenon in the cortex upon stimulus onset^36^. We found that mutual information (MI) about the occurrence of the cue peaked during the consumption window, around 3 to 4 s after the presentation of the tone (Fig. 3d). Interestingly, neurons from the acute fluoxetine condition with the lowest MI values, also showed the smallest changes in firing rate during the consumption window. This suggests that changes in the firing rate during the execution of the task are related to the information conveyed by the population of neurons. Taken together, these results state that mPFC differentially encodes stimulus information as a function of both, the time elapsed from the cue, and 5-HT bioavailability, the effect being consistent across a wide range of timescales.

Mean noise correlation, across all pairs of simultaneously recorded neurons, was positive and weak, as expected for cortical neurons (reported values are in the range between 0.01 to 0.2)^37, 38^. We focused on the significant differences between averaged values for treatments and found a diminished mean correlation due to fluoxetine treatment (Fig. 2c). Although noise correlation can be regarded as a measure of redundancy in a neural code^34^, it does not always imply a reduction in coding capacity^39^. Indeed, we found that the dependence of entropy with noise correlation is disrupted by fluoxetine treatment (Fig. 3b), and this effect is consistent for timescales ranging from 50 to 500 ms (Fig. 3a).

By comparing pairwise and single neuron entropy, we showed that chronic fluoxetine induced a less redundant codification of information. The redundancy observed for pairs of neurons may have a greater effect at the whole population level, which could impair the efficiency of the neural code^40^. Although the decrease in redundancy may be related to a reduced correlation in neuronal activity, this does not necessarily suggest that drug treatment induces a less robust encoding about the occurrence of a reward-predicting stimulus. However, fluoxetine administration produced an impairment in the robustness of stimulus coding, noticed by a faster decay of the MI computed on surrogate data where neuron activation states were corrupted. The dominant source of electrical noise in neurons is the channel noise, which produces fluctuations in membrane potential large enough to affect the timing of action potentials^41^. It has been shown that the information capacity carried out by a spike train is sensitive to the proportion of ion channels present in the neuronal membrane^42^. In this regard, fluoxetine has been reported to be a potent blocker of potassium (K^+^), sodium (Na^+^), and calcium (Ca^2+^) channels^43^. In pyramidal neurons of the PFC the 5-HT_1A_ receptor is abundantly expressed, and is coupled to a G-protein gated inwardly-rectifying K^+^ channel^44^, which produces a hyperpolarizing effect. In contrast, the 5-HT_2A_ receptor, localized in the pyramidal neurons and interneurons, increases intracellular Ca^2+^, and it inhibits calcium-activated afterhyperpolarization currents^45^. Moreover, 5-HT_2A_ stimulation depolarizes fast spiking interneurons via suppression of inward rectifying K^+^ channel^46^. The selective activation and inhibition of ion channel currents by 5-HT and fluoxetine in mPFC could influence, not only the spike timing, but also the action potential initiation in pyramidal neurons, which affect robust encoding by a mechanism of threshold adaptation to input currents^47^.

The state adopted by a network of neurons is strongly dependent on changes in the local recurrent and feedforward synaptic connections^48^. Fluoxetine and its metabolites can act outside of the mPFC and it has been shown to have short and long term effects on the activity of monoaminergic systems^49, 50^. The working of PFC is highly sensitive to neuromodulator concentrations within a very narrow range^7^. It has been proved that fluoxetine increases 5-HT, dopamine (DA) and norepinephrine (NE) concentration in the mPFC^51, 52^. Dopaminergic neurons fire with reward-predicting cues and in a previous work of our group we demonstrated that the ventral tegmental area (VTA) contributes critically to enhance information coding about stimuli in the mPFC of rats performing a GO/NoGO task^53^. Simultaneous recordings of single-unit activity in the mPFC and the DRN of behaving animals are needed to probe the interplay between those areas on robust encoding of reward-related stimulus.

A computational model of PFC showed that 5-HT modulates the performance of spatial working memory through 5-HT_1A_ and 5-HT_2A_ receptors^54^. Moreover, in this model high 5-HT concentrations result in an easily distracted network^55^. Although this task is clearly different from the one analyzed here, our results showed that the information codified in the mPFC is less robust to noise when fluoxetine is administered. In turn, it is expected that a less robust network will be more susceptible to change its activity pattern upon the occurrence of a distractor. In terms of decision-making, a reduction in robustness in the mPFC can be counterproductive when it is necessary to retain information in memory^56^. On the other hand, a less robust network may be advantageous when behavioural flexibility is required, such as when task rules change unexpectedly^57^. There is evidence that 5-HT in PFC has a critical role for flexible response during changes in stimulus-reward contingencies^58^. In this regard, we have shown in a previous study that fluoxetine treatment retarded acquisition but accelerated extinction in an operant conditioning task in rats treated with the same drug dose used in the present work^59^. Nevertheless, behavioral flexibility and stability depend on monoaminergic neurotransmitter levels in the PFC and other areas such as the striatum^8, 60^, and as mentioned above fluoxetine could act through other pathways to mediate the observed effect. Further experiments will be required to clarify the neuronal circuits involved.

In summary, in this work we have shown how systemic fluoxetine treatment affects the information encoding of reward-predicting stimuli in the neural population of the mPFC. To our knowledge, this is the first study in quantifying the effect of a SSRI on the encoding capability of mPFC, contributing to a better understanding of the serotonergic modulation on mPFC during reward-driven learning, whose abnormal functioning underlies cognitive and behavioural characteristics of several neuropsychiatric disorders^61^. We conclude that the systemic increase in endogenous 5-HT bioavailability induces in the mPFC neuronal population a state of low neuronal firing activity, a less redundant encoding capacity and also a less robust encoding of the information about the reward-predictive stimulus. The precise neural mechanism underlying these phenomena is the focus of future work.

## Materials and methods

All experimental procedures involving animals were approved by the Committee of Animal Care and Ethics from the Instituto de Biología y Medicina Experimental-Consejo Nacional de Investigaciones Científicas y Técnicas (IByME-CONICET) and were implemented in compliance with the ARRIVE guidelines in agreement with the recommendations of Directive 2010/63 / EU (ANNEX I) and of “Guide for the Care and Use of Laboratory Animals”, 8th edition, 2010, NRC, EE. USA (ANNEX II).

### Animals

Five male Long Evans rats from the bioterium of IByME were used for the experiments described below. Two months old rats of weight around 300 g were individually housed in stainless steel boxes (40 × 22 × 20 cm) in a light/dark cycle of 12 h, starting the light phase at 8 a.m., and the room temperature was fixed at 22 ± 1 °C. Food and water supply was modified according to the experimental requirements.

### Drug

Fluoxetine (Gador, AR) was suspended on tap water and was orally administered with a feeding needle 3 h before the beginning of each training session to allow systemic absorption.

### Surgery

Surgical procedures were performed as previously described^28^. Rats were deeply anesthetized with a single I.P. dose of ketamine (100 mg/kg) and xylazine (10 mg/kg). The proper state of anaesthesia was tested by the absence of the paw reflex. Throughout the surgery, eyes were covered with ointment to prevent drying. Body temperature was measured by a rectal probe and held constant at 37 °C using a controlled heating pad. The head fur was shaved and the skull was exposed to clearly locate Bregma. One hole of 2 mm in diameter was drilled in the skull at mPFC coordinate (AP =  + 2.7 mm, L =  + 0.5 mm, Bregma as zero). A 3 mm diameter by 4 mm deep plastic cylindrical recording chamber was positioned around the hole. Two stainless-steel screws were positioned in each of the parietal bones (4 screws in total), after being disinfected in ethanol 70%. Finally, the fixing device was held in place, and was cemented to the screws and the recording chamber with dental acrylic. The recording chamber was filled with antibiotic solution (neomycin 3.5 mg/ml, polymyxin B 5000 UI, gramicidin USP 0.025 mg; OFTAL 3, Holliday–Scott, AR), and sealed with a cotton cap. Immediately after surgery rats were subcutaneously injected with 1 mg/kg of the analgesic Meloxicam (Mobic, Boehringer Ingelheim, AR). During postoperative, rats were treated with antibiotics (0.05 mg/ml Enrofloxacin; Floxacin, Afford, AR) and analgesics (Tramadol 5%, 3 drops per 100 ml of water; Finadiet, AR) dissolved in the tap water and placed into the drinking bottle for a recovery period of 7 days. The recording chamber was constantly examined and the antibiotic solution and cotton cap were changed daily to avoid potential infections.

### Electrodes and data acquisition

Electrodes were manufactured with wires of nickel–chromium (12 µm diameter; Kanthal Palm Coast, USA) twisted together with a stirrer, at a constant speed, in order to obtain a tetrode arrangement. This arrangement was introduced into a stainless steel cannula (250 μm in diameter) to give it rigidity. Impedance at the tip of the tetrode was adjusted at values between 0.5 and 0.8 MΩ by gold electrodeposition (gold solution from CIMA Argentina). We introduced two tetrode braids per cannula, and these cannulas were glued together with cyanoacrylate. Tetrodes were grounded with a copper wire clamped to the cannulas and were connected to a pre-amplifier (gain × 10) and finally to the amplifier (gain × 1000). Data was acquired with a National Instruments device at a sampling rate of 30 kHz.

### Experimental procedures

After the post-surgical period, rats were gradually restricted in the daily water intake, until their weight was between 80 and 85% of their ad libitum weight. Subsequently, water was delivered at doses of approximately 12 ml per day to maintain the animal in the same weight throughout the experiment. For the first two days rats were allowed to explore the training chamber. In the following days, rats were carefully wrapped in a Velcro cloth to restrict their movements, and were held on a semi-cylindrical plastic bed (7 cm in diameter and 20 cm long) attaching the fixing device to the stereotaxic frame. The restraining time was increased progressively from 10 to 160 min per day, until animals did not show signs of discomfort or stress at the time of starting the training. On the first training session, rats were trained in a classical conditioning task, supplying a drop of water of approximately 0.06 ml randomly between 1 and 2 s after the presentation of a 1 s long auditory tone as a cue, generated by a computer, regardless of the behaviour showed in that time window, followed by a 4 s inter trial interval (ITI).

On the second training day, the operant protocol was initiated. In the operant conditioning task, rats had to protrude the tongue (lick) after the tone (conditioned stimulus, CS), to obtain a drop of water as a reward (unconditioned stimulus, US) during an opportunity window of 2 s after the end of the tone; otherwise, the drop was not administered. In either case, an ITI of 4 s followed. This protocol was implemented in MATLAB (The MathWorks, Inc., USA) and the movements of the tongue were captured by a camera connected to the computer. Water was supplied through a spout connected to a pump controlled by the computer. Once rats reached a learning criterion of at least 80% of conditioned responses in 100 consecutive trials, the mPFC electrophysiological activity was recorded, simultaneously with operant training.

Before each recording session, the cotton plug was removed and 1 ml of 2% lidocaine solution was applied as a local anaesthetic into the recording chambers for about 3 min. Tetrodes, previously disinfected, were attached to a micro-manipulator and introduced into the hole at mPFC coordinate. Tetrodes were descended at a speed rate of 10–20 μm/s until electrophysiological neuronal activity was found in the form of stable spontaneous action potentials. Neuronal activity was sought up to 3 mm in depth from the surface of the meninges, and if it was not found, the training was continued without recording. A recording session had a duration according to the performance of the rat, i.e., the training sessions were concluded when the animal stopped responding to the cue (due to satiety) or when it consumed the maximum daily amount of water allowed to maintain its weight (about 15 ml).

Each animal was trained in at least 10 control sessions (recording and non-recording sessions), without any drug administration, and consecutively in 10 to 15 sessions with fluoxetine systemic administration (10 mg/kg/day). Each training session was carried out at intervals of 1 to 3 days, but without a fixed lapse between recording sessions. The first recording session under the drug treatment was performed on the third day of fluoxetine administration.

### Data analysis

Analysis and statistical tests were implemented in MATLAB (The MathWorks Inc., USA). The first step in the analysis of electrophysiological data consisted in the identification of the neuronal units based on characteristics of the waveforms of the action potentials. The recorded signal was high-pass filtered at 300 Hz to remove the high frequency events. Then a threshold was manually placed, after visual inspection of the entire recording, to identify the spike events that exceed a certain voltage amplitude. Spikes were extracted and grouped according to their attributes, through a graphical analysis of the principal components, in combination with other features of the waveform, as being the amplitude of the peak of each spike^29^. An analysis of correlation between the spike times of all units throughout the recording was carried out and units with a Spearman’s correlation higher than approximately 0.3 were considered to be the same neuron. Then, a raster at 1 ms time resolution was constructed for each recorded session, containing a sequence of zeros (no spike event) and ones (spike event). Rasters were aligned across trials to construct peri-stimulus time histograms (PSHTs), with 0 ms as the time of tone onset. Subsequently, only the neurons with a signal-to-noise ratio (SNR) greater than 4 were taken into account for analysis. The SNR of an identified neuron was calculated as the ratio between the average value of the spikes at the aligned peaks and the standard deviation of the baseline signal (1 ms before the peak).

The firing rate of an identified neuron was computed using a timescale window of 300 ms. The z-score was calculated as the difference between firing rate of each time window and the mean firing rate across time divided by its standard deviation across trials.

The neural activity was transformed into a binary data to reduce the number of possible states that the neural population could adopt in order to avoid a systematic error due to insufficient sampling^30^. This kind of approach captures almost all variability in cortical networks in second order maximum entropy models^31^. For a given trial and a time window within that trial, if the neuron showed a higher firing frequency than its average across trials, its state was set to 1, otherwise it was set to 0. Taking into account a pair of neurons recorded simultaneously, there are four possible states in a given trial and scale, considering the combinations of the individual binary states of the neurons: *11* state (both neurons firing above their mean), *10* and *01* state (only one of the neurons fires more than it average) or *00* (both fire below its average). We calculated measures taken from information theory (IT), computed on the binary data, to gain insight about the codification of information in the population of mPFC neurons. Then, pairwise Shannon entropy (HS) for a pair of neurons *i* and *j* was computed as:

1 $${HS}_{ij}=-\left({p}_{00}log\left({p}_{00}\right)+{p}_{10}log\left({p}_{10}\right)+{p}_{01}log\left({p}_{01}\right)+{p}_{11}log\left({p}_{11}\right)\right)$$

where *p*_*xy*_ is the probability of occurrence of the binary state *xy* measured across trials.

We assessed the redundancy (*R*) in the information encoded in the neural population by computing the mutual information for pairs of simultaneously recorded neurons *i* and *j*:

2 $${R}_{ij}=H{S}_{i}+H{S}_{j}-H{S}_{ij}$$

where *HS*_*ij*_ is the entropy of the pair of neurons *i* and *j*, and *HS*_*i*_, *HS*_*j*_ are the entropies of neurons *i* and *j* respectively.

We also measured the information conveyed by neuron pairs about the occurrence of the stimulus by computing the mutual information (MI) between the neuron pair binary state and a random variable S that coded if the neural state occurred before the cue (S = BC) or after the cue (S = AC):

3 $${MI}_{ij;S}={HS}_{ij}+{HS}_{S}-{HS}_{ij;S}$$

where *HS*_*S*_ is the entropy of the variable *S*, *HS *_*i j*_ is the entropy of the pair of neurons *i* and *j*, and *HS*_(*ij*,*S*)_ is the joint entropy between pair (*i*,*j*) and *S*. Shannon entropy, together with noise correlation, were computed across trials for pairs of neurons recorded simultaneously, along the successive times of the operant task using a fixed time window. The time BC was settled to 1 s before the tone and its width was that of the timescale used.

We also carry out a robustness analysis of the information encoded. Noise was introduced into the binary data by randomly flipping the state of one neuron of a pair (*i*,*j*) with a given probability *p*_*flip*_, and the *MI*_(*ij*,*S*)_ of the pair (*i*,*j*) was computed according to Eq. (3) for several values of *p*_*flip*_. This procedure gave ordered pairs of *p*_*flip*_ and its associated MI, which were fitted with an exponential function: $MI\left({p}_{flip}\right)={MI}_{data}.{e}^{\left(r.{p}_{flip}\right)}$. The y-intercept (*MI*_*data*_*)*, approximates the MI conveyed by the neuron pairs when states are not corrupted by noise. The parameter *r* measures how robust to noise is the information conveyed by a given pair of neurons.

For statistical comparisons between average effects of treatments, we used the Mann–Whitney U test for two independent samples. When multiple comparisons were needed, repeated-measures ANOVA were used followed by multiple *post-hoc* Tukey's range tests comparing groups of treatments by time (*intra-subject* factor), or comparing time windows by condition.

## Supplementary Information

Supplementary Information.
