# Supplementary material for: Information capacity and robustness of encoding in the medial prefrontal cortex are modulated by the bioavailability of serotonin and the time elapsed from the cue during a reward-driven task
Source: Sci Rep. 2021 Jul 6;11:13882. doi: 10.1038/s41598-021-93313-6 (PMC8260631; doi:10.1038/s41598-021-93313-6)
Supplement: Supplementary file 1 — Supplementary Information. [file 41598_2021_93313_MOESM1_ESM.docx]

**Supplementary Table S1.** **Firing profiles of all recorded neurons**. Number and percentage of neurons where the firing rate was higher (excited), lower (inhibited) or non-significantly different (non-responding) compared to basal activity (at -1 s +/- 0.3 s, before cue onset), for several time windows after the tone. The U Mann-Whitney test was implemented for statistical comparisons.

|  | | Tone  (0 to +1 s) | | Opportunity window  (+1 to +2 s) | Opportunity window  (+2 to +3 s) | | ITI  (+3 to +4 s) | |
| --- | --- | --- | --- | --- | --- | --- | --- | --- |
| Control  (n = 117) | excited | | 55 (47%) | 49 (38%) | | 44 (38%) | | 39 (33%) |
|  | inhibited | | 56 (48%) | 36 (31%) | | 26 (22%) | | 23 (20%) |
|  | non-responding | | 6 (5%) | 32 (27%) | | 47 (40%) | | 55 (47%) |
| Acute  Fluoxetine  (n = 76) | excited | | 33 (43%) | 31 (41%) | | 33 (43%) | | 31 (41%) |
|  | inhibited | | 41 (54%) | 36 (47%) | | 28 (37%) | | 22 (29%) |
|  | non-responding | | 2 (3%) | 9 (12%) | | 15 (20%) | | 23 (30%) |
| Chronic  Fluoxetine  (n = 50) | excited | | 26 (52%) | 19 (38%) | | 10 (20%) | | 14 (28%) |
|  | inhibited | | 20 (40%) | 13 (26%) | | 6 (12%) | | 8 (16%) |
|  | non-responding | | 4 (8%) | 18 (36%) | | 34 (68%) | | 28 (56%) |
